# Crystal structure of apatite type Ca_2.49_Nd_7.51_(SiO_4_)_6_O_1.75_

**DOI:** 10.1107/S205698901600089X

**Published:** 2016-01-20

**Authors:** Thu Hoai Le, Neil R. Brooks, Koen Binnemans, Bart Blanpain, Muxing Guo, Luc Van Meervelt

**Affiliations:** a KU Leuven - University of Leuven, Department of Metallurgy and Materials Engineering, Kasteelpark Arenberg 44 - bus 2450, B-3001 Heverlee, Belgium; b KU Leuven - University of Leuven, Department of Chemistry, Celestijnenlaan 200F - bus 2404, B-3001 Heverlee, Belgium

**Keywords:** crystal structure, apatite structure type, calcium rare earth oxide silicate

## Abstract

The title compound, Ca_2+*x*_Nd_8–*x*_(SiO_4_)_6_O_2–0.5*x*_ (*x* = 0.49), was synthesized at 1873 K and rapidly quenched to room temperature. Its structure has been determined using single-crystal X-ray diffraction and compared with results reported using neutron and X-ray powder diffraction from samples prepared by slow cooling. The single-crystal structure from room temperature data was found to belong to the space group *P*6_3_/*m* and has the composition Ca_2.49_Nd_7.51_(SiO_4_)_6_O_1.75_ [dicalcium octa­neodymium hexa­kis­(ortho­silicate) dioxide], being isotypic with natural apatite and the previously reported Ca_2_Nd_8_(SiO_4_)_6_O_2_ and Ca_2.2_Nd_7.8_(SiO_4_)_6_O_1.9_. The solubility limit of calcium in the equilibrium state at 1873 K was found to occur at a composition of Ca_2+*x*_Nd_8–*x*_(SiO_4_)_6_O_2–0.5*x*_, where *x* = 0.49.

## Abstract 2

Single crystals of Ca_2.49_Nd_7.51_(SiO_4_)_6_O_1.75_ have been synthesized from a mixture of Nd_2_O_3_, CaO and SiO_2_ at 1873 K rapidly quenched to room temperature after 24 h.

## Chemical context   

The study of calcium rare earth oxide silicates is important because they are usually observed in nuclear waste along with rare earth silicates. So far, the calcium rare earth oxide silicates of Nd (Fahey & Weber, 1982 ▸; Fahey *et al.*, 1985 ▸), Sm (PDF 29–365; Smith, 1977 ▸), Eu (PDF 29–320; Smith, 1977 ▸), Gd (PDF 28–212; Smith, 1976 ▸), Tb (PDF 38–256; Lacout, 1986 ▸), and Ce (Skakle *et al.*, 2000 ▸) have been studied. Fahey & Weber *et al.* (1982 ▸) and Fahey *et al.* (1985 ▸) published the structure and stoichiometry limits of the Ca_2+*x*_Nd_8–*x*_(SiO_4_)_2–0.5*x*_ system using X-ray and neutron powder diffraction. In that study, the samples were synthesized at 1523 or 1873 K and cooled at a rate of 250 K per hour. However, such a slow cooling process may lead to undesired modifications of the obtained specimens since the solubility of calcium does not remain constant but decreases with decreasing temperature. This problem is avoided in the present work by rapid quenching of the Ca_2+*x*_Nd_8–*x*_(SiO_4_)_6_O_2–0.5*x*_ samples in their equilibrium state at 1873 K to room temperature within a few seconds. Consequently, compositions of the samples can be preserved better.

## Structural commentary   

The single crystal structure determined from room temperature data was found to belong to the space group *P*6_3_/*m* and has the composition Ca_2.49_Nd_7.51_(SiO_4_)_6_O_1.75_ and is isotypic with natural apatite and the previously reported Ca_2_Nd_8_(SiO_4_)_6_O_2_ and Ca_2.2_Nd_7.8_(SiO_4_)_6_O_1.9_ (Fahey & Weber, 1982 ▸; Fahey *et al.*, 1985 ▸). The solubility limit of calcium in the equilibrium state at 1873 K was found to occur at a composition of Ca_2+*x*_Nd_8–*x*_(SiO_4_)_6_O_2–0.5*x*_, where *x* = 0.49.

**Figure 1:**
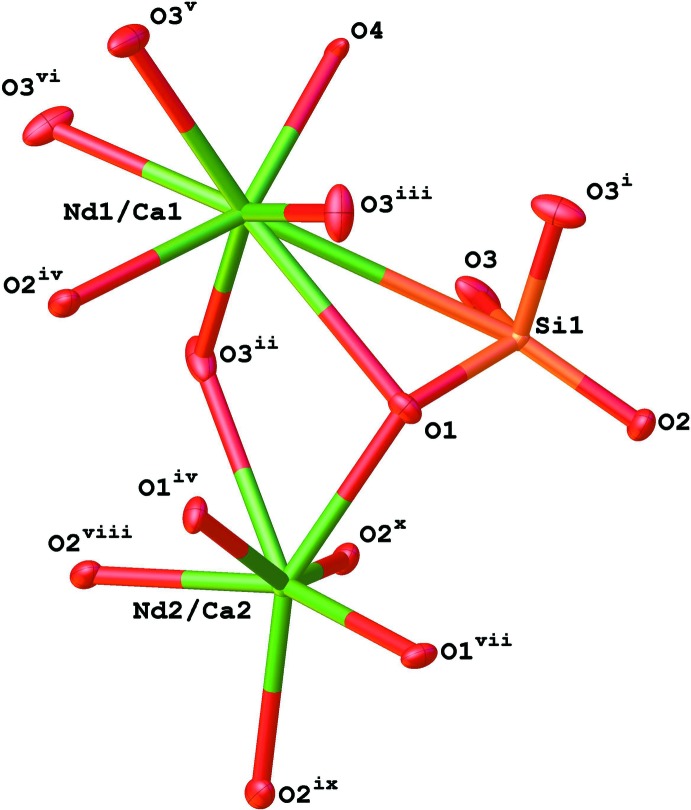
View of the coordination spheres of the Nd/Ca and Si atoms [displacement ellipsoids shown at the 50% probability level; symmetry codes: (i) *x*, *y*, −*z* + 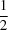; (ii) *y*, −*x* + *y*, −*z*; (iii) *y*, −*x* + *y*, *z* + 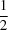; (iv) −*y* + 1, *x* − *y*, *z*; (v) *y* − *x*, −*x*, −*z* + 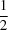; (vi) *y* − *x*, −*x*, *z*; (vii) *y* − *x* + *1*, −*x* + 1, *z*; (viii) *y*, −*x* + *y*, *z* − 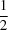; (ix) −*y* + *x* + 1, *x*, *z* − 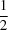; (*x*) −*x* + 1, −*y* + 1, *z* − 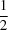].

There are two metal positions in the asymmetric unit of the structure (Fig. 1 ▸) and both contain disordered Nd and Ca ions: Nd1/Ca1 occupies the lower symmetry site 6*h* and Nd2/Ca2 the higher symmetry site 4*f*. The occupancies of these metal sites were refined resulting in 0.887 (5)/0.113 (5) for Nd1/Ca1 and 0.546 (4)/0.454 (4) for Nd2/Ca2. The majority (80%) of calcium is situated at the *4f* site. In the structures of Ca_2_Nd_8_(SiO_4_)_6_O_2_ and Ca_2.2_Nd_7.8_(SiO_4_)_6_O_1.9_, these values are 89 and 73%, respectively (Fahey *et al.*, 1985 ▸). The refined value of the amount of Nd in the structure gives a value of 0.49 for *x* in the equation Ca_2+*x*_Nd_8–*x*_(SiO_4_)_6_O_2–0.5*x*_. For charge-balance purposes, the occupancy of O^2−^ in the structure must be 2 − 0.5*x* or 1.755. Initially, the occupancy of the O^2−^ position O4 in the structure was allowed to refine freely and its value was close to what is required for charge balance; however, it was fixed at 0.146 as the refinement of heavy-atom positions is the most reliable and exact charge balance is required.

The Nd1/Ca1 site is seven coordinate and the Nd/Ca—O bond lengths vary between 2.3909 (19) and 2.721 (3) Å for oxygen atoms of the SiO_4_^2−^ unit but the shortest bond length of 2.2681 (2) Å is to the O^2−^ ion, O4 (Fig. 1 ▸; Table 1 ▸). The Nd2/Ca2 site is nine coordinate and only bonds to SiO_4_^2−^ units with six short distances [Nd—O = 2.4231 (17), 2.4715 (18) Å] and three long distances [Nd—O = 2.830 (2) Å] (Fig. 1 ▸; Table 1 ▸) are observed. The distances are similar to those reported by Fahey *et al.* (1985 ▸) for the structures of Ca_2_Nd_8_(SiO_4_)_6_O_2_ and Ca_2.2_Nd_7.8_(SiO_4_)_6_O_1.9_ determined by powder X-ray diffraction.

**Figure 2:**
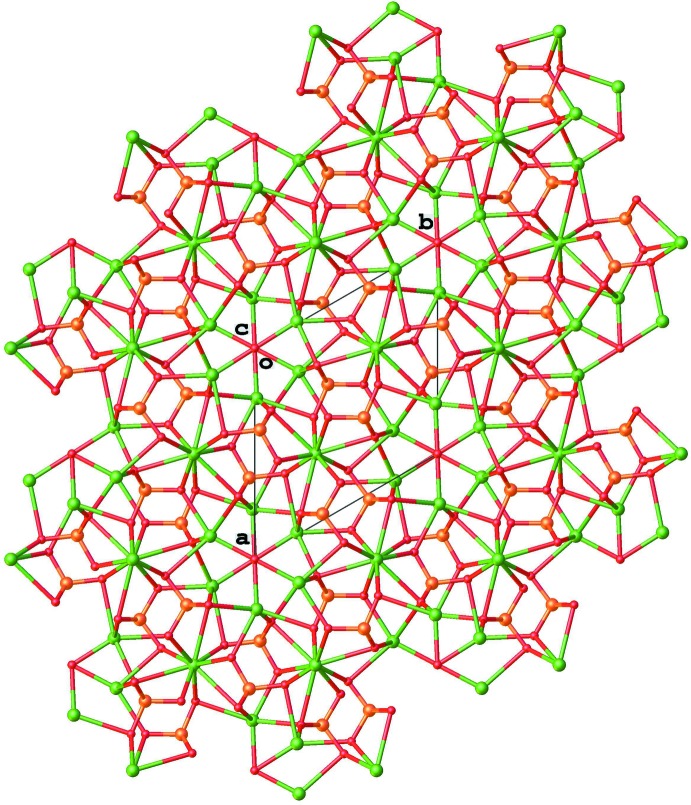
View along the *c* axis of the packing arrangement.

The O4 atom (O^2−^ ion) is coordinated to three different Nd1/Ca1 ions whilst the SiO_4_^4−^ group has eight contacts to different Nd/Ca positions. The O1 atom coordinates one Nd1/Ca1 position and two Nd2/Ca2 positions, the O2 atom coordinates one Nd1/Ca1 position and two Nd2/Ca2 positions and the O3 position coordinates one Nd1/Ca1 and one Nd2/Ca2 positions. These contacts generate the packing, which can be seen viewed down the *c* axis in Fig. 2 ▸.

## Synthesis and crystallization   

A mixture of appropriate amounts of fine powders of Nd_2_O_3_ (99.99%), CaO (99.9%) and SiO_2_ (99.9%) was put into a sealed Pt-20%Rh tube and heated to 1873 K in an argon atmosphere and maintained at that temperature for 24 h. CaO was made by calcination of CaCO_3_ at 1373 K for 12 h. The sample was then quenched in a cold-water bath to give a light-blue crystalline solid, from which a single crystal of the title compound was selected. The sample was further analyzed by EPMA–WDS, giving a composition of 20.2% SiO_2_, 72.1% Nd_2_O_3_ and 7.7% CaO. The converted formula according to the EPMA–WDS result was Ca_2.45_Nd_7.45_Si_6_O_25.775_ (O was calculated).

## Refinement details   

Crystal data, data collection and structure refinement details are summarized in Table 2 ▸. There are two metal positions in the structure and the Nd and Ca ions are disordered on both of these sites. Nd/Ca occupancy on each of the two positions was refined and the occupancy of Nd was found to be 88.7 (5)% for one site and 54.6 (4)% for the other, giving a value of 0.49 for *x* in Ca_2+x_Nd_8–*x*_(SiO_4_)_6_O_2–0.5*x*_. The occupancy of the anionic O atom was fixed at 2 − 0.5*x*. Constraints were applied so that the Nd and Ca on the same site had identical positional and displacement parameters.

## Supplementary Material

Crystal structure: contains datablock(s) I. DOI: 10.1107/S205698901600089X/ru2066sup1.cif

Structure factors: contains datablock(s) I. DOI: 10.1107/S205698901600089X/ru2066Isup2.hkl

CCDC reference: 1447637

Additional supporting information:  crystallographic information; 3D view; checkCIF report

## Figures and Tables

**Table 1:** Selected bond lengths (Å)

Nd1—O1	2.721 (3)	Nd2—O2^iv^	2.4715 (18)
Nd1—O2^i^	2.463 (3)	Nd2—O3^v^	2.830 (2)
Nd1—O3^ii^	2.3909 (19)	Si1—O1	1.621 (3)
Nd1—O3^iii^	2.547 (2)	Si1—O2	1.623 (3)
Nd1—O4	2.2681 (2)	Si1—O3^vi^	1.629 (2)
Nd2—O1^i^	2.4231 (17)		

Symmetry codes: (i) 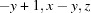; (ii) 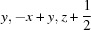; (iii) 
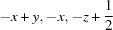
; (iv) 
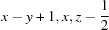
; (v) 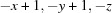; (vi) 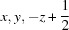.

**Table 2:** Experimental details

Crystal data	
Chemical formula	Ca_2.49_Nd_7.51_(SiO_4_)_6_O_1.75_
*M*_r_	1763.24
Crystal system, space group	Hexagonal, *P*6_3_/*m*
Temperature (K)	298
*a*, *c* (Å)	9.5507 (3), 7.0513 (3)
*V* (Å^3^)	557.03 (3)
*Z*	1
Radiation type	Mo *K*α
μ (mm^−1^)	18.18
Crystal size (mm)	0.05 × 0.05 × 0.05

Data collection	
Diffractometer	Agilent SuperNova (single source at offset, Eos detector)
Absorption correction	Multi-scan (*CrysAlis PRO*; Agilent, 2012 ▸)
*T*_min_, *T*_max_	0.717, 1.000
No. of measured, independent and observed [*I* > 2σ(*I*)] reflections	2616, 878, 813
*R*_int_	0.024
(sin θ/λ)_max_ (Å^−1^)	0.821

Refinement	
*R*[*F* ^2^ > 2σ(*F* ^2^)], *wR*(*F* ^2^), *S*	0.019, 0.035, 1.11
No. of reflections	878
No. of parameters	42
Δρ_max_, Δρ_min_ (e Å^−3^)	0.79, −0.86

Computer programs: *CrysAlis PRO* (Agilent, 2012 ▸), *SHELXS97* (Sheldrick, 2008 ▸), *SHELXL2014* (Sheldrick, 2015 ▸), *OLEX2* (Dolomanov *et al.*, 2009 ▸) and *publCIF* (Westrip, 2010 ▸).

## supplementary crystallographic information

### Crystal data

**Table:**

Ca_2.49_Nd_7.51_(SiO_4_)_6_O_1.75_	*D*_x_ = 5.256 Mg m^−^^3^
*M**_r_* = 1763.24	Mo *K*α radiation, λ = 0.71073 Å
Hexagonal, *P*6_3_/*m*	Cell parameters from 1649 reflections
Hall symbol: -P 6c	θ = 4.3–35.5°
*a* = 9.5507 (3) Å	µ = 18.18 mm^−^^1^
*c* = 7.0513 (3) Å	*T* = 298 K
*V* = 557.03 (3) Å^3^	Block, light blue
*Z* = 1	0.05 × 0.05 × 0.05 mm
*F*(000) = 790	

### Data collection

**Table:**

Agilent SuperNova (single source at offset, Eos detector) diffractometer	878 independent reflections
Radiation source: SuperNova (Mo) X-ray Source	813 reflections with *I* > 2σ(*I*)
Mirror monochromator	*R*_int_ = 0.024
Detector resolution: 15.9631 pixels mm^-1^	θ_max_ = 35.7°, θ_min_ = 3.8°
ω scans	*h* = −15→15
Absorption correction: multi-scan (*CrysAlis PRO*; Agilent, 2012)	*k* = −15→15
*T*_min_ = 0.717, *T*_max_ = 1.000	*l* = −11→5
2616 measured reflections	

### Refinement

**Table:**

Refinement on *F*^2^	Primary atom site location: structure-invariant direct methods
Least-squares matrix: full	Secondary atom site location: difference Fourier map
*R*[*F*^2^ > 2σ(*F*^2^)] = 0.019	*w* = 1/[σ^2^(*F*_o_^2^) + (0.0072*P*)^2^ + 0.3232*P*] where *P* = (*F*_o_^2^ + 2*F*_c_^2^)/3
*wR*(*F*^2^) = 0.035	(Δ/σ)_max_ = 0.001
*S* = 1.11	Δρ_max_ = 0.79 e Å^−^^3^
878 reflections	Δρ_min_ = −0.86 e Å^−^^3^
42 parameters	Extinction correction: *SHELXL2014* (Sheldrick, 2015), Fc^*^=kFc[1+0.001xFc^2^λ^3^/sin(2θ)]^-1/4^
0 restraints	Extinction coefficient: 0.0062 (2)
4 constraints	

### Special details

**Table:**

Geometry. All e.s.d.'s (except the e.s.d. in the dihedral angle between two l.s. planes) are estimated using the full covariance matrix. The cell e.s.d.'s are taken into account individually in the estimation of e.s.d.'s in distances, angles and torsion angles; correlations between e.s.d.'s in cell parameters are only used when they are defined by crystal symmetry. An approximate (isotropic) treatment of cell e.s.d.'s is used for estimating e.s.d.'s involving l.s. planes.
Refinement. Refinement of *F*^2^ against ALL reflections. The weighted *R*-factor *wR* and goodness of fit *S* are based on *F*^2^, conventional *R*-factors *R* are based on *F*, with *F* set to zero for negative *F*^2^. The threshold expression of *F*^2^ > σ(*F*^2^) is used only for calculating *R*-factors(gt) *etc*. and is not relevant to the choice of reflections for refinement. *R*-factors based on *F*^2^ are statistically about twice as large as those based on *F*, and *R*- factors based on ALL data will be even larger.

### Fractional atomic coordinates and isotropic or equivalent isotropic displacement parameters (Å^2^)

**Table:**

	*x*	*y*	*z*	*U*_iso_*/*U*_eq_	Occ. (<1)
Nd1	0.24279 (2)	0.01102 (2)	0.2500	0.00756 (7)	0.887 (5)
Ca1	0.24279 (2)	0.01102 (2)	0.2500	0.00756 (7)	0.113 (5)
Nd2	0.6667	0.3333	−0.00110 (5)	0.00906 (10)	0.546 (4)
Ca2	0.6667	0.3333	−0.00110 (5)	0.00906 (10)	0.454 (4)
Si1	0.37185 (11)	0.40114 (11)	0.2500	0.0077 (2)	
O1	0.4886 (3)	0.3232 (3)	0.2500	0.0127 (5)	
O2	0.4707 (3)	0.5974 (3)	0.2500	0.0144 (5)	
O3	0.2528 (2)	0.3424 (3)	0.0659 (3)	0.0209 (5)	
O4	0.0000	0.0000	0.2500	0.0141 (10)	0.88

### Atomic displacement parameters (Å^2^)

**Table:**

	*U*^11^	*U*^22^	*U*^33^	*U*^12^	*U*^13^	*U*^23^
Nd1	0.00740 (10)	0.00713 (10)	0.00733 (9)	0.00301 (7)	0.000	0.000
Ca1	0.00740 (10)	0.00713 (10)	0.00733 (9)	0.00301 (7)	0.000	0.000
Nd2	0.00913 (12)	0.00913 (12)	0.00892 (15)	0.00456 (6)	0.000	0.000
Ca2	0.00913 (12)	0.00913 (12)	0.00892 (15)	0.00456 (6)	0.000	0.000
Si1	0.0069 (4)	0.0079 (4)	0.0085 (4)	0.0039 (3)	0.000	0.000
O1	0.0116 (12)	0.0187 (13)	0.0123 (12)	0.0108 (10)	0.000	0.000
O2	0.0120 (12)	0.0085 (11)	0.0222 (14)	0.0049 (9)	0.000	0.000
O3	0.0158 (9)	0.0382 (13)	0.0131 (9)	0.0166 (9)	−0.0042 (7)	−0.0098 (8)
O4	0.0052 (12)	0.0052 (12)	0.032 (3)	0.0026 (6)	0.000	0.000

### Geometric parameters (Å, º)

**Table:**

Nd1—Nd1^i^	3.9284 (3)	Si1—Nd2^viii^	3.2527 (8)
Nd1—Nd1^ii^	3.9284 (3)	Si1—Nd2^xi^	3.2527 (8)
Nd1—Nd2^iii^	4.0666 (3)	Si1—Ca2^xi^	3.2527 (8)
Nd1—Si1	3.2877 (9)	Si1—Ca2^viii^	3.2527 (8)
Nd1—Si1^i^	3.1738 (9)	Si1—O1	1.621 (3)
Nd1—O1	2.721 (3)	Si1—O2	1.623 (3)
Nd1—O2^iv^	2.463 (3)	Si1—O3^xii^	1.629 (2)
Nd1—O3^v^	2.3909 (19)	Si1—O3	1.629 (2)
Nd1—O3^vi^	2.3909 (19)	O1—Nd2^iii^	2.4230 (17)
Nd1—O3^i^	2.547 (2)	O1—Ca2^iii^	2.4230 (17)
Nd1—O3^vii^	2.547 (2)	O2—Nd1^iii^	2.463 (3)
Nd1—O4	2.2681 (2)	O2—Ca1^iii^	2.463 (3)
Nd2—Si1^vi^	3.2527 (8)	O2—Nd2^viii^	2.4715 (18)
Nd2—Si1^viii^	3.2527 (8)	O2—Nd2^xi^	2.4715 (18)
Nd2—Si1^ix^	3.2527 (8)	O2—Ca2^xi^	2.4715 (18)
Nd2—O1^iii^	2.4231 (17)	O2—Ca2^viii^	2.4715 (18)
Nd2—O1^iv^	2.4231 (17)	O3—Nd1^ii^	2.547 (2)
Nd2—O1	2.4230 (17)	O3—Nd1^xiii^	2.3909 (19)
Nd2—O2^viii^	2.4715 (18)	O3—Ca1^xiii^	2.3909 (19)
Nd2—O2^ix^	2.4715 (18)	O3—Ca1^ii^	2.547 (2)
Nd2—O2^vi^	2.4715 (18)	O3—Nd2^viii^	2.830 (2)
Nd2—O3^x^	2.830 (2)	O3—Ca2^viii^	2.830 (2)
Nd2—O3^viii^	2.830 (2)	O4—Nd1^ii^	2.2681 (2)
Nd2—O3^vi^	2.830 (2)	O4—Nd1^i^	2.2681 (2)
Si1—Nd1^ii^	3.1738 (9)	O4—Ca1^ii^	2.2681 (2)
Si1—Ca1^ii^	3.1738 (9)	O4—Ca1^i^	2.2681 (2)
			
Nd1^ii^—Nd1—Nd1^i^	60.0	O3^viii^—Nd2—Si1^ix^	92.88 (4)
Nd1^ii^—Nd1—Nd2^iii^	103.981 (6)	O3^vi^—Nd2—Si1^viii^	92.88 (4)
Nd1^i^—Nd1—Nd2^iii^	150.673 (5)	O3^vi^—Nd2—Si1^vi^	30.06 (4)
Si1—Nd1—Nd1^ii^	51.249 (17)	O3^x^—Nd2—Si1^viii^	123.63 (4)
Si1^i^—Nd1—Nd1^ii^	113.889 (17)	O3^vi^—Nd2—O3^viii^	117.44 (2)
Si1^i^—Nd1—Nd1^i^	53.889 (17)	O3^x^—Nd2—O3^vi^	117.45 (2)
Si1—Nd1—Nd1^i^	111.249 (17)	O3^x^—Nd2—O3^viii^	117.45 (2)
Si1—Nd1—Nd2^iii^	58.326 (14)	Nd1^ii^—Si1—Nd1	74.86 (2)
Si1^i^—Nd1—Nd2^iii^	134.034 (14)	Nd1^ii^—Si1—Nd2^viii^	81.18 (2)
Si1^i^—Nd1—Si1	165.14 (2)	Nd1^ii^—Si1—Nd2^xi^	81.18 (2)
O1—Nd1—Nd1^ii^	80.66 (5)	Nd1^ii^—Si1—Ca2^xi^	81.18 (2)
O1—Nd1—Nd1^i^	140.66 (5)	Nd1^ii^—Si1—Ca2^viii^	81.18 (2)
O1—Nd1—Nd2^iii^	35.26 (3)	Ca1^ii^—Si1—Nd1	74.86 (2)
O1—Nd1—Si1^i^	165.45 (5)	Ca1^ii^—Si1—Nd1^ii^	0.000 (8)
O1—Nd1—Si1	29.41 (5)	Ca1^ii^—Si1—Nd2^xi^	81.18 (2)
O2^iv^—Nd1—Nd1^i^	120.15 (6)	Ca1^ii^—Si1—Nd2^viii^	81.18 (2)
O2^iv^—Nd1—Nd1^ii^	179.85 (6)	Ca1^ii^—Si1—Ca2^xi^	81.18 (2)
O2^iv^—Nd1—Nd2^iii^	75.88 (5)	Ca1^ii^—Si1—Ca2^viii^	81.18 (2)
O2^iv^—Nd1—Si1	128.60 (6)	Nd2^xi^—Si1—Nd1	139.386 (19)
O2^iv^—Nd1—Si1^i^	66.27 (6)	Nd2^viii^—Si1—Nd1	139.386 (19)
O2^iv^—Nd1—O1	99.18 (8)	Nd2^xi^—Si1—Nd2^viii^	65.31 (2)
O2^iv^—Nd1—O3^i^	71.01 (7)	Nd2^xi^—Si1—Ca2^viii^	65.31 (2)
O2^iv^—Nd1—O3^vii^	71.01 (7)	Ca2^xi^—Si1—Nd1	139.386 (19)
O3^v^—Nd1—Nd1^i^	110.42 (6)	Ca2^viii^—Si1—Nd1	139.386 (19)
O3^i^—Nd1—Nd1^i^	58.32 (5)	Ca2^xi^—Si1—Nd2^xi^	0.000 (10)
O3^v^—Nd1—Nd1^ii^	94.99 (5)	Ca2^viii^—Si1—Nd2^viii^	0.000 (10)
O3^vi^—Nd1—Nd1^i^	110.42 (6)	Ca2^xi^—Si1—Nd2^viii^	65.31 (2)
O3^i^—Nd1—Nd1^ii^	109.12 (5)	Ca2^xi^—Si1—Ca2^viii^	65.31 (2)
O3^vii^—Nd1—Nd1^ii^	109.12 (5)	O1—Si1—Nd1	55.53 (10)
O3^vii^—Nd1—Nd1^i^	58.32 (5)	O1—Si1—Nd1^ii^	130.39 (10)
O3^vi^—Nd1—Nd1^ii^	94.99 (5)	O1—Si1—Ca1^ii^	130.39 (10)
O3^vi^—Nd1—Nd2^iii^	94.52 (6)	O1—Si1—Nd2^xi^	136.87 (6)
O3^i^—Nd1—Nd2^iii^	112.89 (5)	O1—Si1—Nd2^viii^	136.87 (6)
O3^vii^—Nd1—Nd2^iii^	146.38 (5)	O1—Si1—Ca2^viii^	136.87 (6)
O3^v^—Nd1—Nd2^iii^	42.90 (6)	O1—Si1—Ca2^xi^	136.87 (6)
O3^v^—Nd1—Si1	77.25 (5)	O1—Si1—O2	113.16 (14)
O3^vi^—Nd1—Si1^i^	106.70 (5)	O1—Si1—O3	111.34 (9)
O3^i^—Nd1—Si1^i^	30.67 (4)	O1—Si1—O3^xii^	111.34 (9)
O3^i^—Nd1—Si1	145.64 (5)	O2—Si1—Nd1^ii^	116.45 (10)
O3^vii^—Nd1—Si1^i^	30.67 (4)	O2—Si1—Nd1	168.69 (10)
O3^v^—Nd1—Si1^i^	106.70 (5)	O2—Si1—Ca1^ii^	116.45 (10)
O3^vii^—Nd1—Si1	145.64 (5)	O2—Si1—Nd2^viii^	47.71 (6)
O3^vi^—Nd1—Si1	77.25 (5)	O2—Si1—Nd2^xi^	47.70 (6)
O3^v^—Nd1—O1	70.49 (5)	O2—Si1—Ca2^xi^	47.70 (6)
O3^i^—Nd1—O1	146.95 (5)	O2—Si1—Ca2^viii^	47.71 (6)
O3^vi^—Nd1—O1	70.49 (5)	O2—Si1—O3^xii^	107.49 (10)
O3^vii^—Nd1—O1	146.95 (5)	O2—Si1—O3	107.49 (10)
O3^vi^—Nd1—O2^iv^	84.96 (5)	O3^xii^—Si1—Nd1^ii^	52.89 (7)
O3^v^—Nd1—O2^iv^	84.96 (5)	O3^xii^—Si1—Nd1	78.94 (9)
O3^vii^—Nd1—O3^i^	61.28 (9)	O3—Si1—Nd1^ii^	52.89 (7)
O3^vi^—Nd1—O3^vii^	77.13 (4)	O3—Si1—Nd1	78.94 (9)
O3^vi^—Nd1—O3^i^	136.63 (7)	O3—Si1—Ca1^ii^	52.89 (7)
O3^v^—Nd1—O3^vii^	136.63 (7)	O3^xii^—Si1—Ca1^ii^	52.89 (7)
O3^v^—Nd1—O3^i^	77.13 (4)	O3^xii^—Si1—Nd2^xi^	60.46 (9)
O3^v^—Nd1—O3^vi^	137.40 (11)	O3—Si1—Nd2^xi^	111.52 (8)
O4—Nd1—Nd1^i^	30.0	O3^xii^—Si1—Nd2^viii^	111.52 (8)
O4—Nd1—Nd1^ii^	30.0	O3—Si1—Nd2^viii^	60.46 (9)
O4—Nd1—Nd2^iii^	130.005 (6)	O3^xii^—Si1—Ca2^xi^	60.46 (9)
O4—Nd1—Si1	81.249 (17)	O3^xii^—Si1—Ca2^viii^	111.52 (8)
O4—Nd1—Si1^i^	83.888 (17)	O3—Si1—Ca2^xi^	111.52 (8)
O4—Nd1—O1	110.66 (5)	O3—Si1—Ca2^viii^	60.46 (9)
O4—Nd1—O2^iv^	150.15 (6)	O3^xii^—Si1—O3	105.63 (15)
O4—Nd1—O3^vi^	104.58 (5)	Nd2^iii^—O1—Nd1	104.33 (7)
O4—Nd1—O3^vii^	83.45 (5)	Nd2—O1—Nd1	104.33 (7)
O4—Nd1—O3^i^	83.45 (5)	Nd2^iii^—O1—Nd2	93.89 (9)
O4—Nd1—O3^v^	104.58 (5)	Ca2^iii^—O1—Nd1	104.33 (7)
Si1^vi^—Nd2—Si1^viii^	93.628 (14)	Ca2^iii^—O1—Nd2	93.89 (9)
Si1^ix^—Nd2—Si1^viii^	93.628 (14)	Ca2^iii^—O1—Nd2^iii^	0.000 (11)
Si1^ix^—Nd2—Si1^vi^	93.628 (14)	Si1—O1—Nd1	95.06 (11)
O1^iv^—Nd2—Si1^ix^	98.07 (6)	Si1—O1—Nd2	127.73 (7)
O1^iii^—Nd2—Si1^vi^	165.42 (5)	Si1—O1—Nd2^iii^	127.73 (7)
O1^iv^—Nd2—Si1^vi^	94.28 (5)	Si1—O1—Ca2^iii^	127.73 (7)
O1—Nd2—Si1^vi^	98.07 (6)	Nd1^iii^—O2—Nd2^viii^	115.89 (7)
O1^iii^—Nd2—Si1^viii^	98.07 (6)	Nd1^iii^—O2—Nd2^xi^	115.89 (7)
O1^iv^—Nd2—Si1^viii^	165.42 (5)	Nd1^iii^—O2—Ca2^viii^	115.89 (7)
O1^iii^—Nd2—Si1^ix^	94.28 (5)	Nd1^iii^—O2—Ca2^xi^	115.89 (7)
O1—Nd2—Si1^ix^	165.42 (5)	Ca1^iii^—O2—Nd1^iii^	0.000 (9)
O1—Nd2—Si1^viii^	94.28 (5)	Ca1^iii^—O2—Nd2^xi^	115.89 (7)
O1^iii^—Nd2—O1	72.49 (7)	Ca1^iii^—O2—Nd2^viii^	115.89 (7)
O1^iv^—Nd2—O1^iii^	72.49 (7)	Ca1^iii^—O2—Ca2^xi^	115.89 (7)
O1^iv^—Nd2—O1	72.49 (7)	Ca1^iii^—O2—Ca2^viii^	115.89 (7)
O1—Nd2—O2^vi^	125.79 (8)	Nd2^xi^—O2—Nd2^viii^	90.49 (8)
O1^iv^—Nd2—O2^vi^	94.22 (6)	Nd2^xi^—O2—Ca2^viii^	90.49 (8)
O1—Nd2—O2^ix^	153.93 (8)	Nd2^viii^—O2—Ca2^viii^	0.0
O1^iv^—Nd2—O2^viii^	153.93 (8)	Ca2^xi^—O2—Nd2^viii^	90.49 (8)
O1^iii^—Nd2—O2^ix^	94.22 (6)	Ca2^xi^—O2—Nd2^xi^	0.0
O1^iii^—Nd2—O2^vi^	153.93 (8)	Ca2^xi^—O2—Ca2^viii^	90.49 (8)
O1^iv^—Nd2—O2^ix^	125.79 (8)	Si1—O2—Nd1^iii^	122.72 (13)
O1—Nd2—O2^viii^	94.22 (6)	Si1—O2—Ca1^iii^	122.72 (13)
O1^iii^—Nd2—O2^viii^	125.79 (8)	Si1—O2—Nd2^xi^	103.23 (9)
O1^iii^—Nd2—O3^vi^	139.76 (6)	Si1—O2—Nd2^viii^	103.23 (9)
O1—Nd2—O3^viii^	87.87 (7)	Si1—O2—Ca2^xi^	103.23 (9)
O1^iv^—Nd2—O3^x^	68.15 (7)	Si1—O2—Ca2^viii^	103.23 (9)
O1—Nd2—O3^vi^	68.15 (7)	Nd1^xiii^—O3—Nd1^ii^	116.16 (8)
O1^iii^—Nd2—O3^x^	87.87 (7)	Nd1^xiii^—O3—Ca1^ii^	116.16 (8)
O1^iv^—Nd2—O3^viii^	139.76 (6)	Nd1^ii^—O3—Nd2^viii^	101.98 (7)
O1^iv^—Nd2—O3^vi^	87.87 (7)	Nd1^xiii^—O3—Nd2^viii^	101.99 (8)
O1^iii^—Nd2—O3^viii^	68.15 (7)	Nd1^ii^—O3—Ca2^viii^	101.98 (7)
O1—Nd2—O3^x^	139.76 (6)	Nd1^xiii^—O3—Ca2^viii^	101.99 (8)
O2^vi^—Nd2—Si1^ix^	64.81 (6)	Ca1^ii^—O3—Nd1^ii^	0.000 (14)
O2^ix^—Nd2—Si1^viii^	64.81 (6)	Ca1^xiii^—O3—Nd1^xiii^	0.0
O2^ix^—Nd2—Si1^vi^	98.63 (5)	Ca1^xiii^—O3—Nd1^ii^	116.16 (8)
O2^ix^—Nd2—Si1^ix^	29.07 (6)	Ca1^xiii^—O3—Ca1^ii^	116.16 (8)
O2^viii^—Nd2—Si1^viii^	29.07 (6)	Ca1^ii^—O3—Nd2^viii^	101.98 (7)
O2^viii^—Nd2—Si1^ix^	98.63 (5)	Ca1^xiii^—O3—Nd2^viii^	101.99 (8)
O2^viii^—Nd2—Si1^vi^	64.81 (6)	Ca1^ii^—O3—Ca2^viii^	101.98 (7)
O2^vi^—Nd2—Si1^vi^	29.07 (6)	Ca1^xiii^—O3—Ca2^viii^	101.99 (8)
O2^vi^—Nd2—Si1^viii^	98.63 (5)	Ca2^viii^—O3—Nd2^viii^	0.000 (14)
O2^vi^—Nd2—O2^viii^	75.14 (6)	Si1—O3—Nd1^xiii^	141.68 (11)
O2^vi^—Nd2—O2^ix^	75.14 (6)	Si1—O3—Nd1^ii^	96.43 (9)
O2^ix^—Nd2—O2^viii^	75.14 (6)	Si1—O3—Ca1^xiii^	141.68 (11)
O2^vi^—Nd2—O3^viii^	125.24 (6)	Si1—O3—Ca1^ii^	96.43 (9)
O2^vi^—Nd2—O3^x^	66.19 (7)	Si1—O3—Nd2^viii^	89.48 (10)
O2^viii^—Nd2—O3^viii^	58.84 (7)	Si1—O3—Ca2^viii^	89.48 (10)
O2^ix^—Nd2—O3^x^	58.84 (7)	Nd1^i^—O4—Nd1^ii^	120.0
O2^vi^—Nd2—O3^vi^	58.85 (7)	Nd1—O4—Nd1^ii^	120.0
O2^viii^—Nd2—O3^x^	125.24 (6)	Nd1—O4—Nd1^i^	120.0
O2^ix^—Nd2—O3^viii^	66.19 (7)	Nd1—O4—Ca1^ii^	120.0
O2^ix^—Nd2—O3^vi^	125.24 (6)	Nd1^i^—O4—Ca1^ii^	120.0
O2^viii^—Nd2—O3^vi^	66.19 (7)	Nd1—O4—Ca1^i^	120.0
O3^vi^—Nd2—Si1^ix^	123.63 (4)	Ca1^i^—O4—Nd1^i^	0.000 (16)
O3^viii^—Nd2—Si1^viii^	30.06 (4)	Ca1^ii^—O4—Nd1^ii^	0.000 (9)
O3^x^—Nd2—Si1^ix^	30.06 (4)	Ca1^i^—O4—Nd1^ii^	120.0
O3^x^—Nd2—Si1^vi^	92.88 (4)	Ca1^i^—O4—Ca1^ii^	120.0
O3^viii^—Nd2—Si1^vi^	123.63 (4)		

Symmetry codes: (i) −*x*+*y*, −*x*, −*z*+1/2; (ii) −*y*, *x*−*y*, *z*; (iii) −*x*+*y*+1, −*x*+1, −*z*+1/2; (iv) −*y*+1, *x*−*y*, *z*; (v) *y*, −*x*+*y*, *z*+1/2; (vi) *y*, −*x*+*y*, −*z*; (vii) −*x*+*y*, −*x*, *z*; (viii) −*x*+1, −*y*+1, −*z*; (ix) *x*−*y*+1, *x*, *z*−1/2; (x) *x*−*y*+1, *x*, −*z*; (xi) *x*−*y*, *x*, *z*+1/2; (xii) *x*, *y*, −*z*+1/2; (xiii) *x*−*y*, *x*, *z*−1/2.
